# Micro Gesture Recognition with Multi-Dimensional Feature Fusion and CQ-MobileNetV3 Using FMCW Radar

**DOI:** 10.3390/s25226949

**Published:** 2025-11-13

**Authors:** Wei Xue, Rui Wang, Jianyun Wei, Li Liu

**Affiliations:** 1 School of Automation, China University of Geosciences, Wuhan 430074, China; iswangrui@cug.edu.cn (R.W.); jianyunw@cug.edu.cn (J.W.); liliu@cug.edu.cn (L.L.); 2 Hubei Key Laboratory of Advanced Control and Intelligent Automation for Complex Systems, Wuhan 430074, China; 3 Engineering Research Center of Intelligent Technology for Geo-Exploration, Ministry of Education, Wuhan 430074, China

**Keywords:** micro gesture recognition, frequency-modulated continuous wave (FMCW) radar, multi-dimensional feature fusion, deep neural network

## Abstract

Radar-based gesture recognition technology has gained increasing attention in the context of contactless human–computer interaction (HCI). Micro gestures have smaller motion amplitudes and shorter duration compared with traditional gestures, which increases the difficulty of motion feature extraction. In addition, improving recognition accuracy while maintaining low computational and storage costs is also a challenge. In this paper, a micro gesture recognition method combining multi-dimensional feature fusion and a lightweight CQ-MobileNetV3 network is proposed. For feature extraction, the range–time map, velocity–time map, and angle–time map of gestures are first constructed. Then, normalization and adaptive filtering are performed to refine the three maps. Finally, the three refined maps are fused to form a range–velocity–angle–time map, which can accurately describe the motion characteristics of gestures. For recognition, a lightweight CQ-MobileNetV3 network is designed. First, the network structure of MobileNetV3 is optimized to reduce computational complexity. Then, the improved convolutional block attention module (CBAM) and the improved self-attention (SA) module are constructed and integrated into different bottleneck blocks to improve recognition accuracy. A series of experiments are conducted with a 77 GHz frequency-modulated continuous wave (FMCW) radar. The results indicate that CQ-MobileNetV3 achieves a recognition accuracy of 97.16% for 14 micro gestures, with a parameter count of 0.207 M and a computational complexity of 0.027 GFLOPs, surpassing several other deep neural networks.

## 1. Introduction

Gestures are an important form of human body language, with the advantages of simplicity, efficiency and universality [1]. At present, gesture recognition has been applied in various fields of human–computer interaction (HCI), such as smart home [2], virtual reality [3], intelligent driving [4], medical assistance [5,6], and sign language translation [7].

To date, different types of sensors have been applied to gesture recognition, including wearable sensors [8], visual sensors [9,10], ultrasonic sensors [11], Wi-Fi devices [12] and radar sensors [13,14]. Wearable sensors can precisely acquire gesture motion information, demonstrating good recognition performance. However, they require the user to wear devices and can be inconvenient, which limits their application scenarios. Visual sensors extract gesture feature information through cameras or depth cameras, but their recognition performance may decrease under insufficient lighting conditions, and they may also lead to user privacy leakage. Ultrasonic sensors use ultrasonic waves to detect gesture motion information, but they are prone to diffraction and the speed of sound propagation. Wi-Fi devices utilize the channel state information of Wi-Fi signals to perceive gesture movements. They are not affected by lighting conditions but are susceptible to the multipath effect. Radar sensors are not influenced by weather and light, which can prevent user privacy leakage. Among various types of radars, a frequency-modulated continuous wave (FMCW) radar operating in the millimeter wave band has the merits of low cost, small size and high measurement accuracy and is considered the most promising non-contact gesture recognition technology.

Extensive studies have been conducted on gesture recognition based on radar. These studies have mainly focused on gesture feature extraction methods and recognition algorithms. Gesture features generally include the distance, velocity (Doppler), angle, and point clouds. Recognition algorithms mainly consist of machine learning algorithms and deep learning algorithms. Common machine learning algorithms include support vector machines (SVMs), artificial neural networks (ANNs), and K-nearest neighbors (KNNs). Li et al. [15] extracted six features of gestures, including azimuth, elevation, and Doppler information, and employed a shallow ANN for classification, achieving an average recognition accuracy of 93.3% for six gestures. Zhang et al. [16] extracted two Doppler features and used SVM for recognition, obtaining a recognition accuracy of 93.6% for four gestures. Rashid et al. [17] extracted Doppler spectra, used principal component analysis (PCA) to decrease the spectral feature dimension, and performed classification using a KNN classifier, achieving an accuracy of nearly 100% for four gestures.

Deep learning algorithms mainly include convolutional neural networks (CNNs), long short-term memory (LSTM) networks, and Transformer networks. Wang et al. [18] employed range-Doppler images (RDIs) as features and utilized a CNN with LSTM units for recognition, obtaining a recognition rate of 87% for 11 gestures. Wang et al. [19] extracted the projection features from the range-Doppler map (RDM) and used a CNN to recognize six micro gestures, achieving a recognition rate of 98.06%. Ali et al. [20] extracted time–velocity and time–angle diagrams and utilized a CNN to recognize six micro gestures, obtaining an average accuracy of 95%. Wang et al. [21] extracted the range–Doppler map (RDM) and range–angle map (RAM) and employed a 3D-CNN for recognizing eight gestures, achieving an accuracy of 93.12%. Yu et al. [22] extracted RDIs and range–angle images (RAIs) and employed a CNN-LSTM network to classify 12 gestures, obtaining an accuracy of 94.67%. Wu et al. [23] extracted range–time maps (RTMs) and angle–time maps (ATMs) and designed a lightweight CNN model for feature fusion and classification for 11 gestures, achieving an accuracy of 97.53%. Yang et al. [24] obtained the motion trajectory of gestures from point clouds and used a single-layer LSTM network for recognition, achieving an average recognition accuracy of 98.2% for 10 air-digit-writing gestures. Song et al. [25] extracted the range–time map (RTM), Doppler–time map (DTM), azimuth–time map (ATM), and elevation–time map (ETM) and used DenseNet with a convolutional block attention module (CBAM) for recognition, obtaining a recognition accuracy of 99.03% for 12 micro gestures. Li et al. [26] extracted the RDM and used ResNet50 for recognition, achieving a recognition accuracy of 98.02% for four gestures. Li et al. [27] obtained trajectory points of gestures and converted them into images, then used Xception for recognition, achieving a recognition accuracy of 99.6% for 10 digital gestures. Jin et al. [28] constructed sparse time–Doppler–range features from range–Doppler maps and utilized a dual-flow Transformer network for recognition, obtaining an accuracy of 99.17% for six letter gestures.

From the aforementioned research, it can be seen that machine learning algorithms are suitable for processing low-dimensional features and are generally applied in recognition scenarios with fewer gesture types. Deep learning algorithms are suitable for processing high-dimensional features with stronger feature extraction abilities, making them widely used in gesture recognition. In deep learning algorithms, the gesture features mainly consist of two types: temporal features and sequential features. Temporal features such as RDIs and RAIs reflect the motion information of gestures at a certain moment. Generally, an LSTM network is required to process the temporal features of multiple moments and extract time sequential information for classification. However, the computational complexity of the LSTM network increases linearly with sequence length. Sequential features such as RTMs, DTMs, and ATMs describe the motion information of the entire gesture duration and can be recognized using a CNN. Therefore, sequence features are adopted in this work. Different from the traditional macro gestures involving hand-level movements, micro gestures mainly involve finger-level movements, with smaller motion variations and shorter duration [29]. The similarity between different micro gestures increases the difficulty of feature extraction. To improve gesture recognition performance, existing methods generally extract multiple features to characterize the motion of gestures. However, multiple features increase the dimensionality of information and may lead to information redundancy. Meanwhile, multiple features need to be fused in the network, increasing the computational complexity. Therefore, how to accurately recognize multiple micro gestures with fewer features and lightweight networks is also a challenge. To address this challenge, this study proposes a method combining the fusion of multi-dimensional features and a lightweight network. The main contributions of this work are as follows.

(1) A feature extraction algorithm that integrates multi-dimensional features is proposed. First, the range–time map (RTM), velocity–time map (VTM), and angle–time map (ATM) are constructed from the raw data. Then, the three maps are refined using normalization and adaptive filtering. Finally, the refined RTM, VTM, and ATM are fused to construct the range–velocity–angle–time map (RVATM). RVATM can fully describe the motion information of micro gestures, with low feature dimensionality and less information redundancy.
(2) A lightweight micro gesture recognition network CQ-MobileNetV3 is proposed based on the MobileNetV3 network. First, several convolution layers and bottleneck blocks are deleted, and the expansion size of bottleneck blocks is reduced, which can reduce computational and storage requirements. Then, an improved convolutional block attention module (CBAM) is constructed, in which the convolution operations of some modules are simplified, and the simpler activation function is adopted to reduce computational complexity. Meanwhile, an improved self-attention (SA) module is designed, in which grouped query technology is employed to reduce computational burden. Finally, the two improved attention modules are integrated into different bottleneck blocks to enhance feature extraction capabilities with little addition in computational complexity. The combination of three improvements enables the network to remain lightweight while maintaining high recognition accuracy.
(3) An experimental platform is designed, and a micro gesture dataset is constructed to evaluate the proposed method. A dataset containing 14 micro gestures in three scenarios is constructed using TI’s IWR1443 FMCW radar sensor. The experimental results verify the robustness and superiority of the proposed method.

The rest of the paper is organized as follows. In Section 2, the principle of the FMCW radar is presented. In Section 3, the details of multi-dimensional feature fusion are provided. Section 4 describes the lightweight CQ-MobileNetV3 network. Section 5 gives experimental verification results. In Section 6, the summary of this work is provided.

## 2. Principle of FMCW Radar

### 2.1. System Architecture and Signal Model

**Figure 1:**
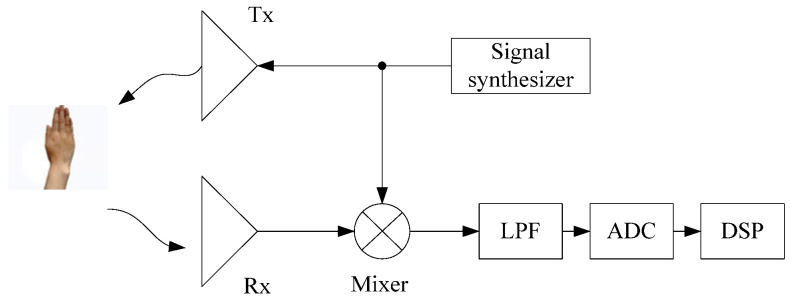
FMCW radar system architecture.

Figure 1 illustrates the architecture of the FMCW radar system [30]. The FMCW signal is generated by the signal synthesizer and transmitted by the transmitting antenna (Tx). The receiving antenna (Rx) is used to receive the target echo. The received signal is mixed with the transmitted signal through a mixer, and after low-pass filtering (LPF), the intermediate frequency (IF) signal is obtained. Finally, an analog-to-digital converter (ADC) is used to sample the IF signal, which is then processed by a digital signal processing (DSP) module.

**Figure 2:**
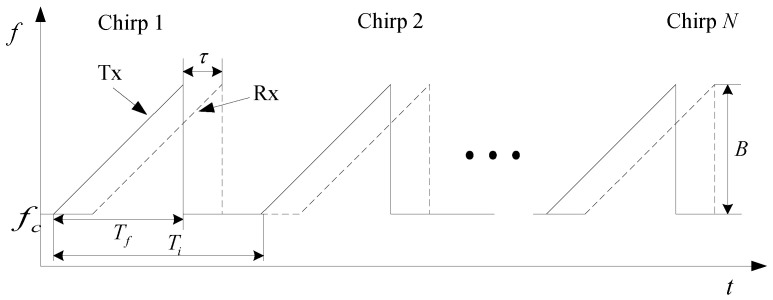
Frequency domain waveform of the transmitted and received signals of FMCW radar.

Generally, FMCW radar transmits multiple chirps to detect the target. The frequency domain waveform of the transmitted and received signals of the FMCW radar is illustrated in Figure 2, where $f_{c}$ is the starting frequency, *B* is the modulation bandwidth, $T_{f}$ is the sweep duration, $T_{i}$ is the chirp period, and τ is the time delay.

The transmitted FMCW signal is given by

(1) $$s_{T}\left(t\right)=A_{T}cos\left[2\pi \left(f_{c}t+\frac{\mu t^{2}}{2}\right)+\varphi_{0}\right]$$

where *A_T_* is the amplitude of the transmitted FMCW signal, $\mu =B/T_{f}$ is the modulation slope, and $\varphi_{0}$ is the initial phase.

The time delay of the echo can be given by

(2) $$\tau =\frac{2(R+vt)}{c}$$

where *c* is the light velocity, and *R* and *v* are the range and velocity of the target, respectively.

Then, the received signal is written as

(3) $$s_{R}(t)=A_{R}cos\left[2\pi \left(f_{c}(t-\tau )+\frac{\mu }{2}{\left(t-\tau \right)}^{2}\right)+\varphi_{0}\right]$$

where $A_{R}$ is the amplitude of the received signal.

The transmitted and the received signals are mixed and low-pass-filtered to obtain the IF signal, which can be given by

(4) $$s_{IF}=A_{IF}cos\left\{2\pi \left(f_{c}\tau +\mu t\tau -\frac{\mu }{2}\tau^{2}\right)\right\}$$

Substituting (2) into (4), the IF signal can be rewritten as

(5) $$s_{R}(t)=A_{R}cos\left[2\pi \left(f_{c}(t-\tau )+\frac{\mu }{2}{\left(t-\tau \right)}^{2}\right)+\varphi_{0}\right]$$

As $2\mu vt^{2}/c$ and $4\mu {\left(R+vt\right)}^{2}/2c^{2}$ can be neglected, the IF signal can be simplified to

(6) $$\begin{array}{ll} s_{IF} & \approx A_{IF}cos\left\{2\pi \left(\frac{2\mu Rt}{c}+f_{c}\frac{2(R+vt)}{c}\right)\right\}\\ & =A_{IF}cos\left\{2\pi \left(\frac{2\mu R}{c}+\frac{2v}{\lambda }\right)t+\frac{4\pi R}{\lambda }\right\} \end{array}$$

where $\lambda =c/f_{c}$ is the wavelength.

Then, the frequency and phase of the IF signal are represented as

(7) $$f_{IF}=\frac{2\mu R}{c}+\frac{2v}{\lambda }$$

(8) $$\varphi_{IF}=\frac{4\pi R}{\lambda }$$

### 2.2. Range Measurement

Due to the slow velocity of gestures, the second term is much smaller than the first term and can be neglected in (7), so the IF frequency can be given by

(9) $$f_{IF}=\frac{2\mu R}{c}$$

Then, the target range can be given by

(10) $$R=\frac{f_{IF}c}{2\mu }$$

Because the duration of chirp is $T_{f}$, the resolution of the IF frequency can be defined as

(11) $$\Delta f_{IF}=\frac{1}{T_{f}}$$

Therefore, the range resolution is represented as

(12) $$R_{res}=\frac{\Delta f_{IF}c}{2\mu }=\frac{c}{2B}$$

### 2.3. Velocity Measurement

In (7), the variation in velocity has little effect on the IF frequency, thus the velocity cannot be obtained from the IF frequency. However, the target motion can lead to obvious changes in the phase between adjacent chirps, especially for millimeter wave signals.

The phase difference between the IF signals corresponding to two adjacent chirps can be expressed as

(13) $$\Delta \varphi_{IF}=\frac{4\pi vT_{i}}{\lambda }$$

Then, the target velocity is given by

(14) $$v=\frac{\lambda \Delta \varphi_{IF}}{4\pi T_{i}}$$

To obtain the maximum unambiguous velocity, $\left|\Delta \varphi_{IF}\right|\leq \pi$ should be satisfied. Therefore, the maximum velocity is

(15) $$v_{max}=\frac{\lambda }{4T_{i}}$$

when using *N* chips for velocity measurement, the minimum value of phase difference is 2π/N, and the velocity resolution can be obtained as

(16) $$v_{res}=\frac{\lambda }{2NT_{i}}$$

### 2.4. Angle Measurement

**Figure 3:**
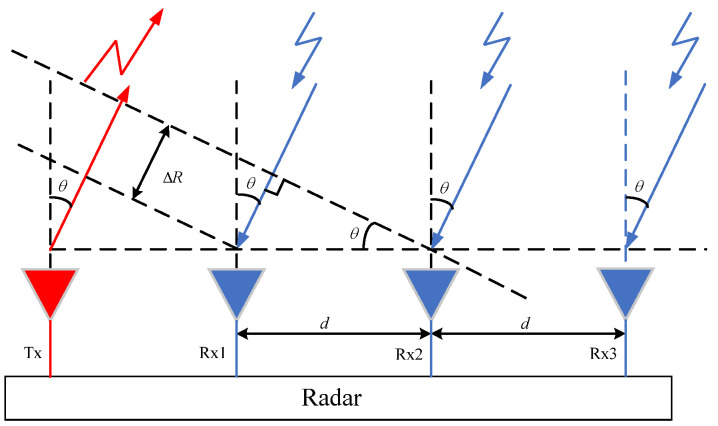
Angle measurement using antenna arrays.

Angle measurement requires at least two receiving antennas. Due to the far-field conditions in the experimental environment, the paths of the echoes to different receiving antennas can be considered as parallel incidence. The principle of angle measurement using antenna arrays is shown in Figure 3.

The range difference for signals of two adjacent receiving antennas can be represented as

(17) ΔR=dsinθ

where *d* is the spacing of adjacent receiving antennas, and *θ* is the incident angle.

Then, the phase difference for signals of two adjacent receiving antennas can be written as

(18) $$\Delta \varphi =\frac{2\pi \Delta R}{\lambda }=\frac{2\pi dsin\theta }{\lambda }$$

The incident angle is given by

(19) $$\theta =sin^{-1}\left(\frac{\lambda \Delta \varphi }{2\pi d}\right)$$

## 3. Multi-Dimensional Feature Fusion

**Figure 4:**
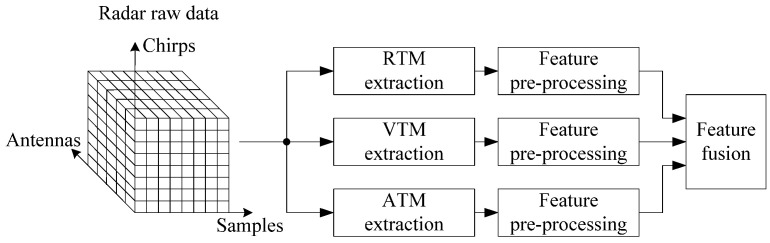
Multi-dimensional feature fusion flow chart.

Figure 4 shows the flow chart of multi-dimensional feature fusion. First, the sampling data of the IF signal is rearranged into the format of Samples × Chirps × Antennas. Subsequently, RTM and VTM are extracted from the multi-frame data of one antenna, where one frame contains multiple chirps. Simultaneously, ATM is obtained from the multi-frame data of multiple antennas. Then, the three maps ATM, VTM, and ATM are refined using feature preprocessing. Finally, the three refined maps are fused to construct the range–velocity–angle–time map (RVATM).

### 3.1. RTM and VTM Extraction

The extraction of RTM and VTM needs multi-frame data of one antenna. First, the two-dimensional data in one frame is processed using 2-D fast Fourier transform (2-D FFT) to obtain the range-Doppler map (RDM). Then, the coherent accumulation is performed separately in the range and Doppler dimensions of the RDM to obtain one-dimensional range information and one-dimensional velocity information of this frame. Finally, RTM and VTM are generated through stacking one-dimensional range information and velocity information from multiple frames.

**Figure 5:**
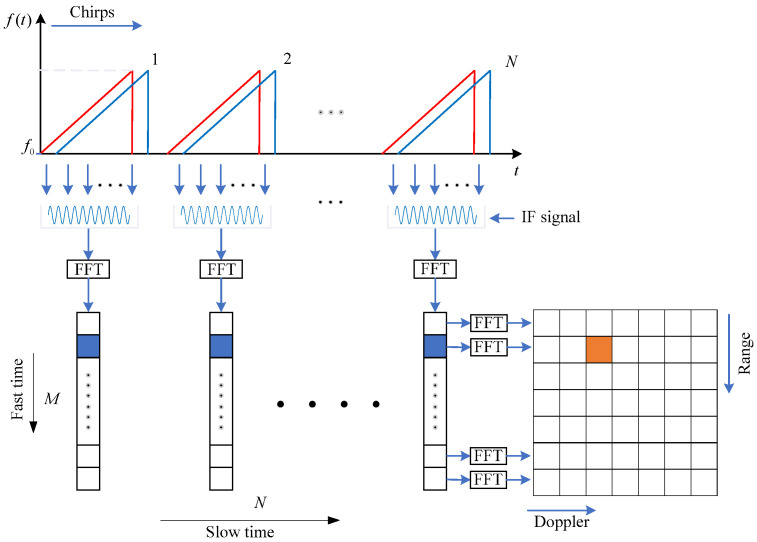
Schematic of RDM generation.

Assume the two-dimensional data matrix in one frame is *T*[*m*, *n*], where 1≤m≤M, 1≤n≤N, one chirp contains *M* samples, and one frame contains *N* chirps. Figure 5 shows the schematic of RDM generation, where the sample dimension is also called the fast time dimension, and the chirp dimension is also called the slow time dimension.

First, the range spectrum is obtained by performing FFT in the fast time dimension, which is represented as

(20) $$R(k,n)=FFT\left[T(m,n)\right]=\sum_{m=1}^{K}T(m,n)exp\left[-j\frac{2\pi }{K}m(k-1)\right]$$

where 1≤k≤K, and *K* is the number of FFT points.

Then, the range–Doppler map is generated by performing FFT in the slow time dimension, which is given by

(21) $$\begin{array}{ll} RD(k,l) & =FFT\left[R(k,n)\right]=FFT2\left[T(m,n)\right]\\ & =\sum_{n=1}^{L}\left(\sum_{m=1}^{K}T(m,n)exp\left[-j\frac{2\pi }{K}m(k-1)\right]\right)exp\left[-j\frac{2\pi }{L}n(l-1)\right] \end{array}$$

where 1≤l≤L, and *L* is the number of FFT points.

Subsequently, one-dimensional range information is obtained by coherent accumulation in the range dimension of RDM, which is given by

(22) $$FR(k)=\sum_{l=1}^{L}RD(k,l)$$

Similarly, one-dimensional velocity information is obtained by correlation accumulation in the Doppler dimension of RDM, which is given by

(23) $$FV(l)=\sum_{k=1}^{K}RD(k,l)$$

Finally, the one-dimensional range and velocity information of multiple frames are stacked to generate RTM and VTM, which can be represented as

(24) $$F_{RTM}=\left[FR_{1}FR_{2}\cdots FR_{NF}\right]$$

(25) $$F_{VTM}=\left[FV_{1}FV_{2}\cdots FV_{NF}\right]$$

where *FR_i_* and *FV_i_* represent the range and velocity information of the *i*th frame, respectively, 1≤i≤NF, and *NF* is the number of frames.

### 3.2. ATM Extraction

The extraction of ATM requires multi-frame data from multiple antennas. Here, the multiple signal classification (MUSIC) algorithm [31] is utilized for angle extraction to obtain higher angular resolution.

Assume the number of receiving antennas is *W* and the incident angles of *D* targets are $\left\{\theta_{1},\theta_{2},\cdots ,\theta_{D}\right\}$, the direction matrix can be written as

(26) $$\begin{array}{ll} A & =\left[\begin{array}{cccc} 1 & 1 & ... & 1\\ e^{-j\frac{2\pi dsin\theta_{1}}{\lambda }} & e^{-j\frac{2\pi dsin\theta_{2}}{\lambda }} & ... & e^{-j\frac{2\pi dsin\theta_{D}}{\lambda }}\\ ... & ... & ... & ...\\ e^{-j(W-1)\frac{2\pi dsin\theta_{1}}{\lambda }} & e^{-j(W-1)\frac{2\pi dsin\theta_{2}}{\lambda }} & ... & e^{-j(W-1)\frac{2\pi dsin\theta_{D}}{\lambda }} \end{array}\right]\\ & =\left[a\left(\theta_{1}\right)a\left(\theta_{2}\right)\cdots a\left(\theta_{D}\right)\right] \end{array}$$

where $a\left(\theta_{i}\right)={\left[1e^{-j\frac{2\pi dsin\theta_{i}}{\lambda }}\cdots e^{-j\left(W-1\right)\frac{2\pi dsin\theta_{i}}{\lambda }}\right]}^{T}$ is the direction vector of the angle $\theta_{i}$, which represents the response of the antennas to the *i*th incident signal.

Then, the received signal of W antennas is represented as

(27) X(t)=A(t)S(t)+N(t)

where *S*(*t*) is the vector of the incident signal, and *N*(*t*) is the vector of noise.

The covariance matrix of the received signal is written as

(28) $$R_{xx}=E[X(t)X{\left(t\right)}^{H}]=AE[SS^{H}]A^{H}+E[NN^{H}]=\sum_{i=1}^{D}\sigma_{i}^{2}a\left(\theta_{i}\right)a^{H}\left(\theta_{i}\right)+\sigma_{n}^{2}I$$

where *H* represents the conjugate transpose operation, $\sigma_{i}^{2}$ and $\sigma_{n}^{2}$ are the power of the ith incident signal and noise, respectively.

Generally, the estimation of covariance matrix is given by

(29) $${\hat{R}}_{xx}=\frac{1}{P}\sum_{i=1}^{P}X(i)X^{H}(i)$$

where *P* is the number of snapshots.

The eigenvalue decomposition of the covariance matrix is given by

(30) $${\hat{R}}_{xx}=U_{s}\Lambda_{s}U_{S}^{H}+U_{N}\Lambda_{N}U_{N}^{H}=U_{s}\Lambda_{s}U_{S}^{H}+\sigma_{n}^{2}U_{N}U_{N}^{H}$$

where $\Lambda_{s}$ and $\Lambda_{N}$ are diagonal matrices corresponding to the signal and noise, respectively, and $U_{S}$ and $U_{N}$ are the eigenvalue matrices of the signal subspace and noise subspace, respectively.

Ideally, $U_{S}$ and $U_{N}$ are orthogonal, and the direction vector in the signal subspace and the noise subspace are also orthogonal, which can be written as

(31) $$a^{H}(\theta )U_{N}=0$$

Therefore, a spatial spectrum is constructed to estimate the angle, which is represented as

(32) $$P(\theta )=\frac{1}{a^{H}(\theta )U_{N}U_{N}^{H}a(\theta )}$$

In the MUSIC algorithm, the angle corresponding to the maximum value of P(θ) is the signal incident direction.

In one frame, the data of one chirp from multiple antennas are used to estimate the covariance matrix and obtain the spatial spectrum. In practical calculation, assuming the step of spatial spectrum search is Δθ, the discrete one-dimensional angle information can be defined as

(33) FA(p)=P(pΔθ)

where *p* is an integer and -π/(2Δθ)≤p≤π/(2Δθ).

Finally, by stacking the one-dimensional angle information of multiple frames, the ATM can be obtained as

(34) $$F_{ATM}=\left[FA_{1}\;FA_{2}\;\cdots \;FA_{NF}\right]$$

where *FA_i_* represents the angle information of the *i*th frame.

### 3.3. Feature Preprocessing

In the measurement, the range variation in the gesture causes the variation in the strength of the IF signal, which further leads to fluctuations in the amplitude of range, velocity, and angle information in each frame. In addition, echoes from other parts of the human body and environmental clutter also result in interference in RTM, VTM, and ATM. To reduce the impact of the above factors, normalization and adaptive filtering are used to process the three maps to improve the quality of feature extraction.

First, normalization is used to reduce the difference in the strength of features between different frames, which can be given by

(35) $$FR1(k)=\frac{FR(k)}{max\left(\left|FR(k)\right|\right)}$$

(36) $$FV1(l)=\frac{FV(l)}{max\left(\left|FV(l)\right|\right)}$$

(37) $$FA1(p)=\frac{FA(p)}{max\left(\left|FA(p)\right|\right)}$$

Subsequently, adaptive filtering is applied to suppress interference, which is calculated as

(38) $$FR2(k)=FR1(k)exp\left(kr\left|k-k_{0}\right|\right)$$

(39) $$FV2(l)=FV1(l)exp\left(kv\left|l-l_{0}\right|\right)$$

(40) $$FA2(p)=FA1(p)exp\left(ka\left|p-p_{0}\right|\right)$$

where *kr*, *kv*, and *ka* are the filter coefficients for range, velocity, and angle, respectively; and *kr* < 0, *kv* < 0, *ka* < 0, *k*_0_, *l*_0_, and *p*_0_ are the positions corresponding to the maximum amplitude of the range, velocity, and angle features in each frame, respectively.

Finally, the preprocessed three maps can be expressed as

(41) $$FP_{RTM}=\left[FR2_{1}FR2_{2}\cdots FR2_{NF}\right]$$

(42) $$FP_{VTM}=\left[FV2_{1}FV2_{2}\cdots FV2_{NF}\right]$$

(43) $$FP_{ATM}=\left[FA2_{1}FA2_{2}\cdots FA2_{NF}\right]$$

where *FR*2*_i_*, *FV*2*_i_*, and *FA*2*_i_* represent the preprocessed range, velocity and angle information of the *i*th frame, respectively.

### 3.4. Feature Fusion

The preprocessed RTM, VTM, and ATM are fused in the color space. Three feature maps are encoded separately into the RGB channels of the image and concatenated to obtain the range–velocity–angle–time map (RVATM), which can be expressed as

(44) $$F_{RVATM}=cat\left[FP_{RTM},FP_{VTM},FP_{ATM}\right]$$

where $cat\left[\cdot \right]$ is the concatenation function. The red, green, and blue channels represent range, velocity, and angle features, respectively.

RVATM integrates multiple motion features into one feature map, reducing feature dimensionality and information redundancy. Using RVATM as the input feature map does not require feature fusion in the network, resulting in lower computational complexity.

## 4. CQ-MobileNetV3 Network

### 4.1. MobileNetV3 Network

MobileNetV3 is a classic lightweight network [32], which integrates depthwise separable convolution, inverted residual structures, and squeeze-and-excitation (SE) attention modules. Depthwise separable convolution divides the standard convolution operation into depthwise (DW) convolution and pointwise (PW) convolution, reducing the demand for computational resources. The inverted residual structure enhances the feature representation capability while reducing the network complexity. The SE attention module extracts important information of channels to improve the feature expression ability.

**Figure 6:**
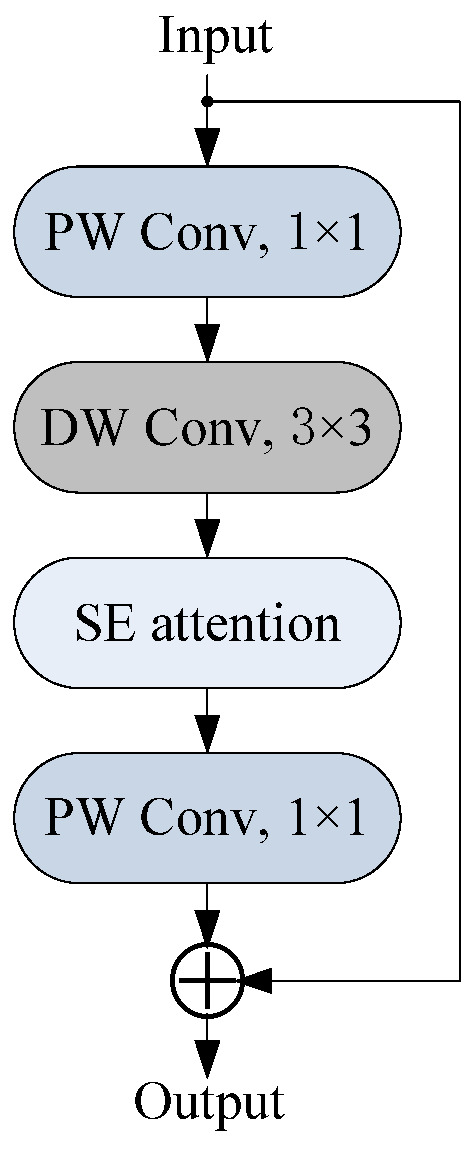
Bottleneck block of MobileNetV3.

The basic unit of MobileNetV3 is the bottleneck block, as shown in Figure 6. First, the input features are dimensionality enhanced using a 1 × 1 PW convolution. Then, a 3 × 3 DW convolution is performed on the dimensionality enhanced features. Next, the features are fed into the SE attention module to obtain attention-weighted features. Finally, the 1 × 1 PW convolution is used to decrease the dimensionality of features, and the output features and initial input features are added as the final output.

**Figure 7:**
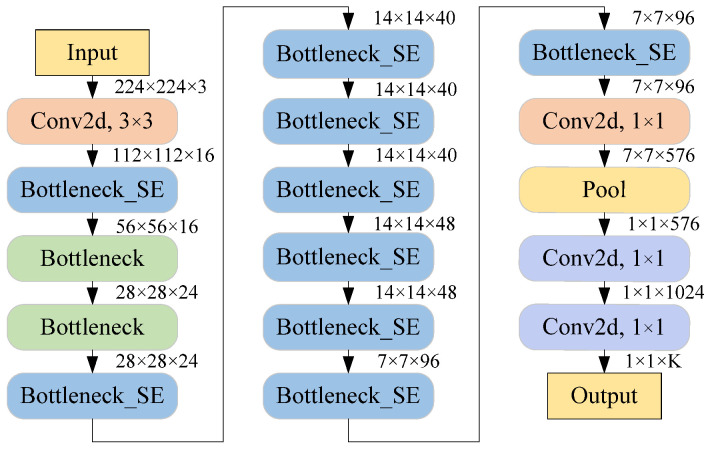
Architecture of MobileNetV3-Small.

In terms of the number of basic units, MobileNetV3 includes two versions, namely MobileNetV3-Large and MobileNetV3-Small. Among them, MobileNetV3-Small is applicable in resource-constrained scenarios and therefore is used as the baseline network. Figure 7 illustrates the architecture of MobileNetV3-Small. Bottleneck_SE represents the bottleneck block with the SE attention module, and Bottleneck represents the bottleneck block without the SE attention module.

In MobileNetV3-Small, a 3 × 3 convolutional layer is first employed for preliminary feature extraction. Subsequently, 11 stacked bottleneck blocks are employed for feature extraction. Next, a 1 × 1 convolution layer is applied to increase the feature dimension, and then global average pooling is performed to obtain the one-dimensional vector feature. Finally, two 1 × 1 convolutional layers map features to probability values for K classes.

### 4.2. Improved CBAM

The SE attention mechanism [33] in MobileNetV3 only considers the importance of each channel and ignores spatial information, which limits its ability to capture the spatial features of scattered regions of interest in gesture feature maps. To overcome the drawback, an improved convolutional block attention module (CBAM) is introduced into MobileNetV3.

**Figure 8:**
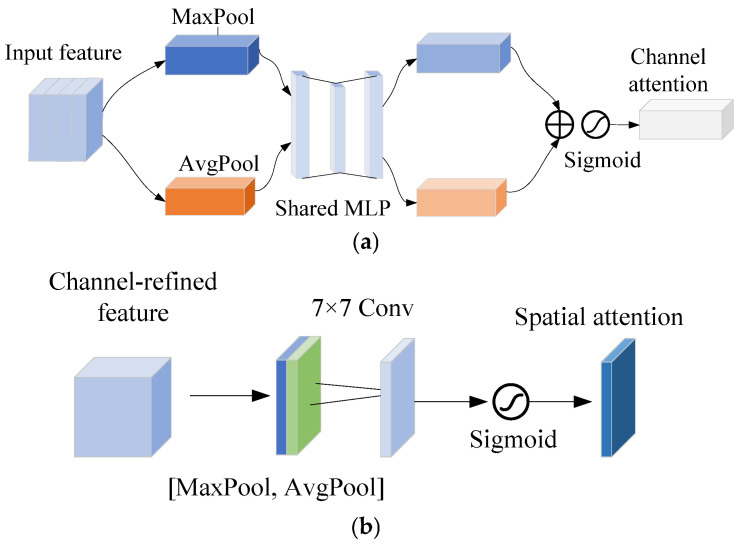
Structure of CBAM: (**a**) CAM; (**b**) SAM.

The structure of CBAM is shown in Figure 8. The CBAM [34] is composed of the channel attention module (CAM) and the spatial attention module (SAM). The input features are first input into CAM to obtain channel-refined features; then, the features are input into SAM to obtain the spatial-refined features.

In CAM, first the input feature F undergoes max pooling and average pooling to obtain two feature descriptors. Then, the two feature descriptors are mapped into two weight vectors by a multi-layer perceptron (MLP). Finally, the two weight vectors are merged and normalized to generate the channel attention weights. The calculation can be given by

(45) $$\begin{array}{c} M_{C}(F)=\sigma \left(MLP\left(MaxPool(F)\right)+MLP\left(AvgPool(F)\right)\right)\\ =\sigma \left(W_{1}\left(W_{0}(F_{max}^{c})\right)+W_{1}\left(W_{0}(F_{avg}^{c})\right)\right) \end{array}$$

where σ is the Sigmoid activation function, and *W*_0_ and *W*_1_ represent the two-layer convolution operations of MLP, respectively.

In SAM, first the channel-refined features are also processed through max pooling and average pooling along the channel dimension to obtain two weight vectors. Subsequently, the two weight vectors pass through a convolutional layer and are normalized to generate spatial attention weights. The calculation is given by

(46) $$M_{S}(F)=\sigma \left(f^{7\times 7}\left(\left[MaxPool(F);AvgPool(F)\right]\right)\right)$$

where $f^{7\times 7}$ denotes the 7 × 7 convolution operation.

**Figure 9:**
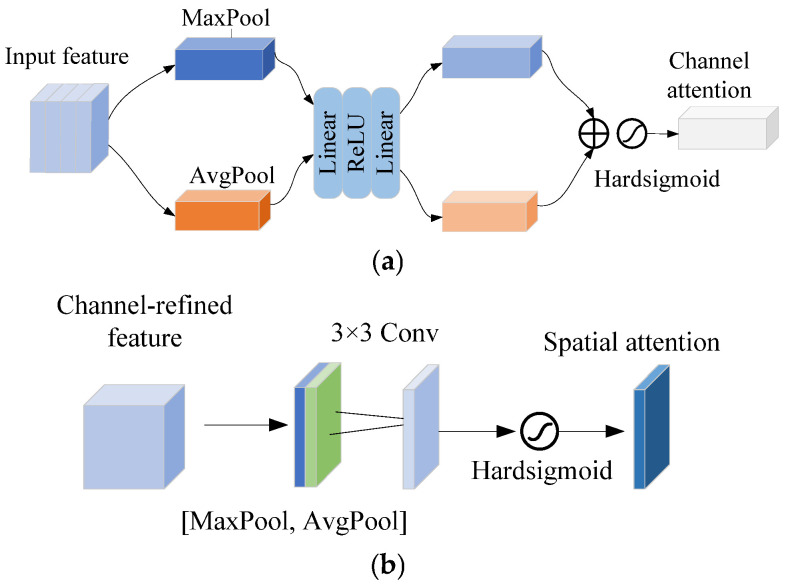
Structure of the improved CBAM: (**a**) improved CAM; (**b**) improved SAM.

Here, two improvements are proposed for CAM to reduce the computational complexity. Figure 9a shows the structure of the improved CAM. First, a two-layer linear transformation is used to replace the convolution operation in MLP. Then, the Hardsigmoid function [35] is used to replace the Sigmoid function. The calculation of improved channel attention can be calculated as

(47) $$M_{C}^{\prime }(F)=Hardsigmoid\left(W_{1}^{\prime }\cdot ReLU\left[W_{0}^{\prime }\cdot \left(MaxPool(F)+AvgPool(F)\right)\right]\right)$$

where $W_{0}^{\prime }$ and $W_{1}^{\prime }$ represent two-layer linear transformations, respectively.

The linear transformation avoids the unnecessary dimension transformations in MLP and can reduce the computational cost.

The Hardsigmoid function is defined as

(48) $$Hardsigmoid(x)=\left\{\begin{array}{ll} 1, & x>3\\ x/6+1/2, & -3\leq x\leq 3\\ 0, & x<-3 \end{array}\right.$$

The Hardsigmoid function is a piecewise linear approximation of the Sigmoid function, avoiding exponential operations, which can improve computational efficiency and reduce resource consumption.

Similarly, two improvements are also proposed for SAM. Figure 9b shows the structure of the improved SAM. First, a 3 × 3 convolution is used to replace the original 7 × 7 convolution. Then, the original Sigmoid function is replaced by the Hardsigmoid function. The calculation of improved spatial attention is given by

(49) $$M_{S}^{\prime }(F)=Hardsigmoid\left(f^{3\times 3}\left(\left[MaxPool(F);AvgPool(F)\right]\right)\right)$$

### 4.3. Improved SA Module

The motion features in the gesture feature map have strong time correlation. The SA mechanism [36] determines the weight of value (*V*) by calculating the similarity between query (*Q*) and key (*K*), which can effectively capture temporal dependence and correlation in the sequence. Therefore, the SA mechanism is also introduced into MobileNetV3.

**Figure 10:**
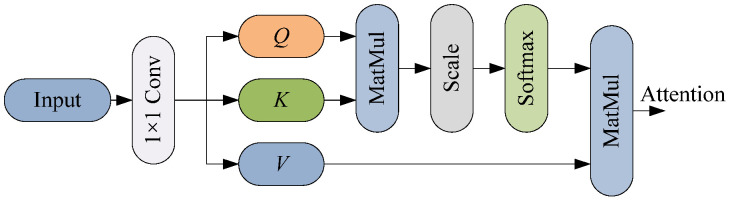
Structure of the SA module.

In gesture recognition, the SA mechanism has two advantages. First, it can capture the correlation between cross-frame gestures by calculating the feature similarity between any two positions. Second, it performs a weighted sum of the value based on the weights obtained from the similarity, enhancing the feature representation of key frames. Figure 10 shows the structure of the SA module.

In Figure 10, *Q*, *K* and *V* are the query, key, and value matrices, respectively. The SA mechanism calculates the attention weights by performing the dot-product operation of *Q*, *K*, and *V*, which can be given by

(50) $$Attention(Q,K,V)=softmax\left(\frac{QK^{T}}{\sqrt{d_{k}}}\right)V$$

where $d_{k}$ represents the dimension of *K*.

Although the SA mechanism has excellent feature expression ability, its computational cost increases linearly with the quadratic of the input feature size. To solve the problem, the grouped query technique is introduced into the calculation of attention. First, group convolution is used to divide the input channels into G groups, and then the attention is calculated separately for each group. Finally, the attention of each group of channels is concatenated. The calculation can be given by

(51) $$GroupAttn(Q,K,V)=\overset{G}{\underset{g=1}{⊕}}softmax\left(\frac{Q_{g}K_{g}^{T}}{\sqrt{d_{k}/G}}\right)V_{g}$$

where *g* is the index of the group, *G* is set to 4, and ⊕ represents the concatenation operation.

Of course, the grouped query technology only focuses on specific keys and values within each group, which may limit the network’s ability to capture global information in some cases.

### 4.4. Optimization of Network Structure

The MobileNetV3-Small network contains 11 bottleneck blocks. Due to the small size of gesture feature maps, excessively deep network layers may lead to unstable gradients. In addition, some bottleneck blocks have a high expansion size, which can also result in redundant calculations.

To address these issues, two optimization strategies are proposed for the network structure. First, two convolution layers and four bottleneck blocks are removed, which can greatly decrease the number of parameters and computational complexity. Second, the expansion size of some bottleneck blocks is reduced to decrease memory consumption. Table 1 lists the optimized network structure.

### 4.5. Overall Network Structure

**Figure 11:**
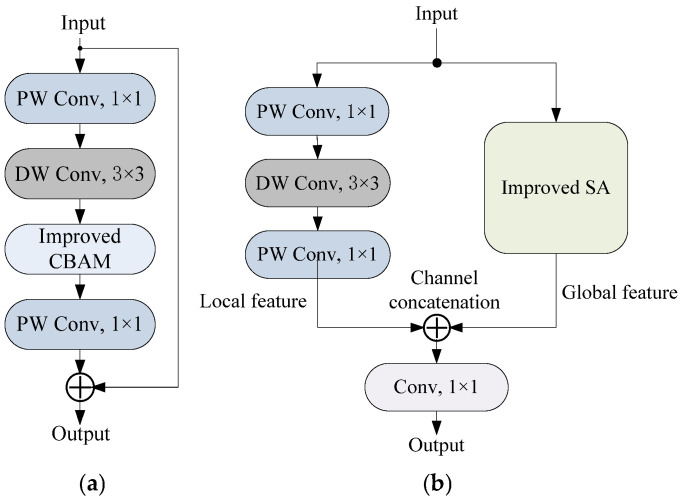
Improved bottleneck blocks: (**a**) Bottleneck_CBAM; (**b**) Bottleneck_SA.

Based on the two improved attention modules, two improved bottleneck blocks are designed, as shown in Figure 11.

Bottleneck_CBAM uses the improved CBAM to replace the SE attention module, which can better extract spatial features in gesture feature maps. Bottleneck_SA utilizes depthwise separable convolution to extract local features, while utilizing the improved SA module to extract global features. Subsequently, channel concatenation is performed on the two features to better preserve local details and global semantics.

**Figure 12:**
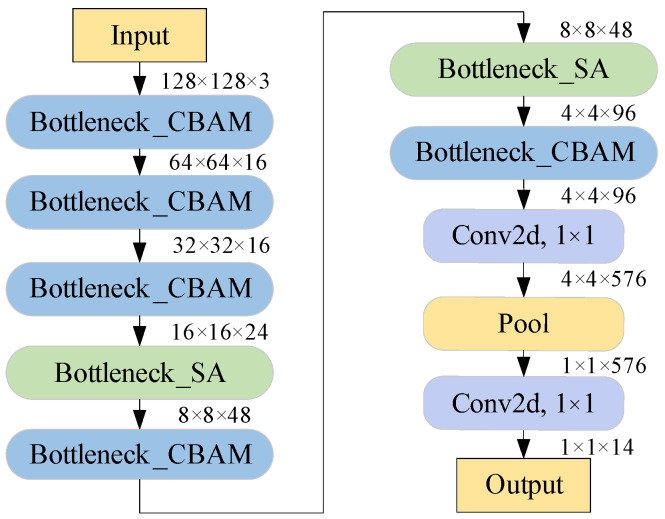
Structure of the CQ-MobileNetV3 network.

According to the optimized network structure and two improved bottleneck blocks, a lightweight CQ-MobileNetV3 network is proposed, the structure of which is shown in Figure 12. The CQ-MobileNetV3 network consists of five Bottleneck_CBAMs, two Bottleneck_SA modules, two 1 × 1 convolutional layers, and one global average pooling module. Due to the high correlation between the computational complexity of the SA module and the size of the input feature map, the Bottleneck_SA module is placed at the deeper layer of the network, which can further reduce the computational cost.

The optimized network structure can significantly reduce the number of parameters and computational complexity. In addition, two types of improved attention modules can enhance recognition accuracy at a low computational cost. Through these three improvements, the proposed network can achieve high recognition accuracy with low computational complexity.

## 5. Experiments and Analysis

### 5.1. Experimental Setup and Parameter Configuration

**Figure 13:**
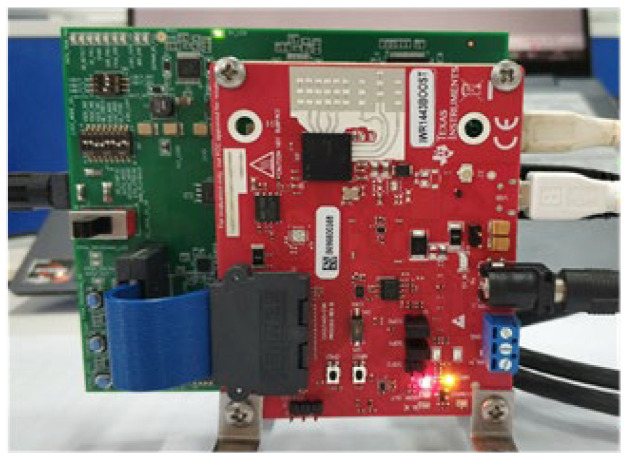
FMCW radar system.

The FMCW radar system for collecting gesture data consists of an IWR1443 radar sensor and a DCA-1000 data acquisition card developed by TI, which is shown in Figure 13. Table 2 lists the parameter configuration of the radar system.

In the experiment, all programs are run on a computer with an Intel I7-10700K CPU with 64 GB RAM and an RTX 4060 GPU with 8 GB of graphic memory. Feature extraction, preprocessing, and feature fusion of radar data are implemented using MATLAB 2017. Deep neural networks are implemented on the PyTorch 1.10 platform using Python 3.10.

### 5.2. Dataset

#### 5.2.1. Data Collection

**Figure 14:**
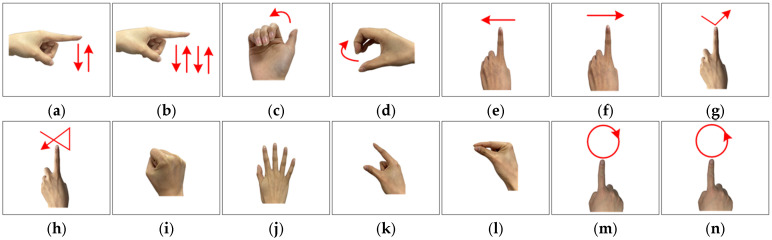
Fourteen gestures: (**a**) click; (**b**) double click; (**c**) beckoning; (**d**) wave; (**e**) sliding left; (**f**) sliding right; (**g**) drawing tick; (**h**) drawing fork; (**i**) palm clench; (**j**) palm open; (**k**) forefinger–thumb open; (**l**) forefinger–thumb close; (**m**) rotating clockwise; (**n**) rotating counterclockwise.

In this study, 14 micro gestures are designed. The detailed movements of the 14 micro gestures are shown in Figure 14. The 14 micro gestures include click, double click, beckoning, wave, sliding left, sliding right, drawing tick, drawing fork, palm clench, palm open, forefinger–thumb open, forefinger–thumb close, rotating clockwise, and rotating counterclockwise.

**Figure 15:**
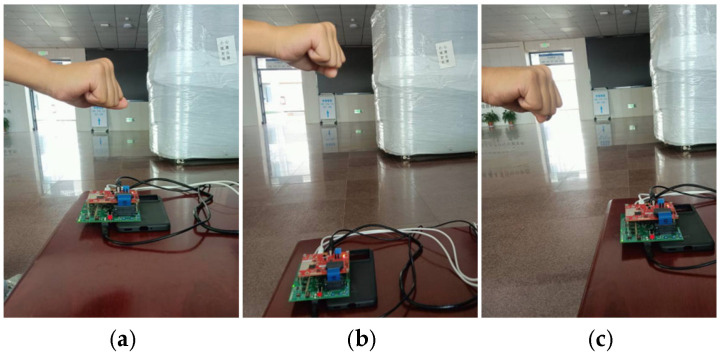
Data collection scenarios: (**a**) Scenario 1; (**b**) Scenario 2; (**c**) Scenario 3.

Figure 15 shows three different data collection scenarios. In all three scenarios, the radar was placed horizontally on a table in a relatively spacious hall. In Scenario 1, the gesture was performed above the radar, at a distance of approximately 0.2 m from the radar. In Scenario 2, the gesture was also performed above the radar, at a distance of about 0.5 m from the radar. In Scenario 3, the gesture was performed above a certain angle perpendicular to the radar, approximately 0.25 m away from the radar.

A total of eight volunteers participated in the gesture data collection. These volunteers consisted of five males and three females. For each gesture, 25 samples were collected from each volunteer in each scenario, and the total sample size was 8400.

#### 5.2.2. Feature Extraction and Preprocessing Results

For RTM and VTM extraction, the RDM is first obtained by performing 2-D FFT on one frame of data collected from one RX antenna. As shown in Table 2, one frame contains 128 chirps, with each chirp containing 64 samples. When performing 2-D FFT, the number of sample points in each chirp is added to 128 by zero padding, resulting in an RDM with a size of 128 × 128. Then, coherent accumulation is performed in the range and Doppler dimensions of RDM to obtain one-dimensional range and velocity information with a size of 128 × 1. Finally, the one-dimensional range and velocity information of 50 frames are stacked along the time dimension to obtain the RTM and VTM with a size of 128 × 50. The range resolution is 0.0234 m, and the maximum range is 3 m. The velocity resolution is 0.0401 m/s, and the maximum velocity is 2.56 m/s.

The 2Tx-4Rx antenna configuration is equivalent to the 1Tx-8Rx antenna units. Therefore, for ATM extraction, the data of one chirp in one frame collected from eight RX antennas is processed using the MUSIC algorithm to obtain the one-dimensional angle information. Here, the step of spatial spectrum search Δθ is set to π/127, and the size of one-dimensional angle information is also 128 × 1. Finally, the one-dimensional angle information of 50 frames is stacked along the time dimension to obtain the ATM with a size of 128 × 50.

In preprocessing, for each frame in RTM, VTM, and ATM, normalization is first performed to reduce the intensity differences between different frames. Then, adaptive filtering is used to suppress interference within each frame of the three feature maps, where the filter coefficients *kr*, *kv*, and *ka* are all set to −0.2.

**Figure 16:**
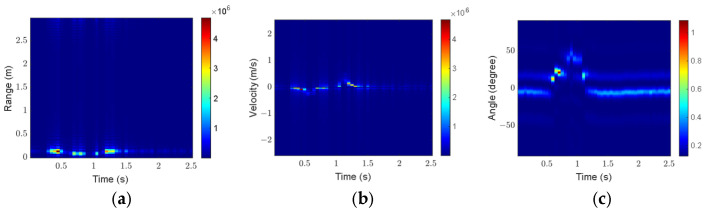
Feature maps of the click gesture before preprocessing in Scenario 1: (**a**) RTM; (**b**) VTM; (**c**) ATM.

**Figure 17:**
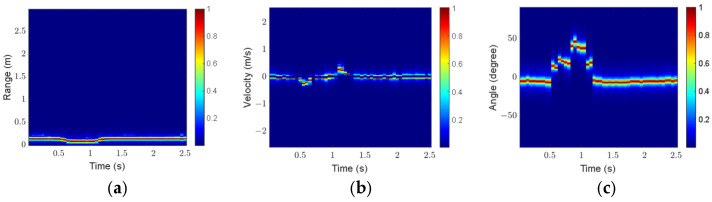
Feature maps of the click gesture after preprocessing in Scenario 1: (**a**) RTM; (**b**) VTM; (**c**) ATM.

The click gesture in Scenario 1 is taken as an example to analyze the influence of preprocessing on feature extraction. Figure 16 and Figure 17 show the three feature maps before and after preprocessing, respectively.

As shown in Figure 16, the gesture echoes in the original RTM and VTM are weak at some moments, and breakpoints occur in the motion trajectories. In addition, some clutter appears in the original ATM. As shown in Figure 17, after preprocessing, the gesture echo distribution in RTM and VTM is more uniform, and the clutter in ATM is well suppressed, which can more accurately describe the motion characteristics of the click gesture.

**Figure 18:**
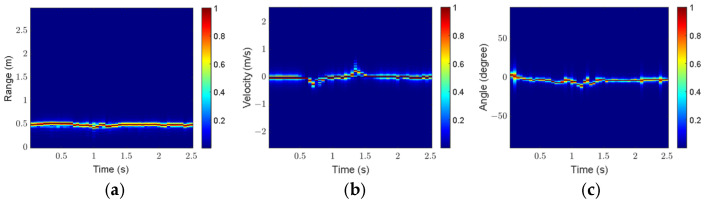
Feature maps of the click gesture after preprocessing in Scenario 2: (**a**) RTM; (**b**) VTM; (**c**) ATM.

**Figure 19:**
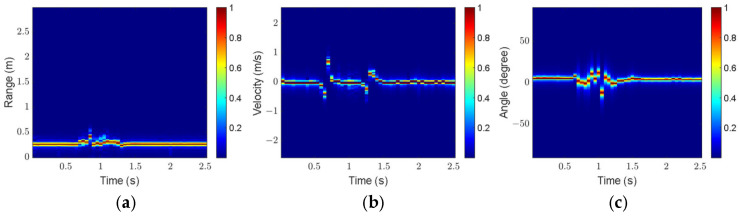
Feature maps of the click gesture after preprocessing in Scenario 3: (**a**) RTM; (**b**) VTM; (**c**) ATM.

For comparison, Figure 18 and Figure 19 present the three feature maps of the click gesture after preprocessing in Scenarios 2 and 3, respectively.

As shown in Figure 17, Figure 18 and Figure 19, the range variation for the click gesture is minimal in the three scenarios, especially for Scenario 3, which has a longer range. The velocity of the click gesture varies significantly and similarly across the three scenarios. The angle changes in the click gesture across the three scenarios are also obvious but different. The differences in the feature maps of the same gesture in different scenarios can effectively increase the diversity of the dataset.

**Figure 20:**
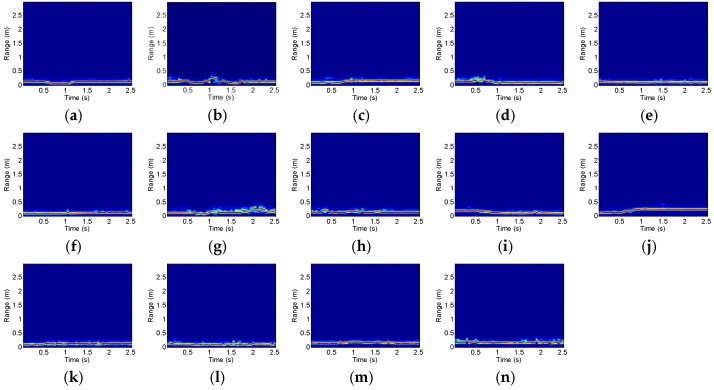
RTMs of 14 gestures after preprocessing: (**a**) click; (**b**) double click; (**c**) beckoning; (**d**) wave; (**e**) sliding left; (**f**) sliding right; (**g**) drawing tick; (**h**) drawing fork; (**i**) palm clench; (**j**) palm open; (**k**) forefinger–thumb open; (**l**) forefinger–thumb close; (**m**) rotating clockwise; (**n**) rotating counterclockwise.

**Figure 21:**
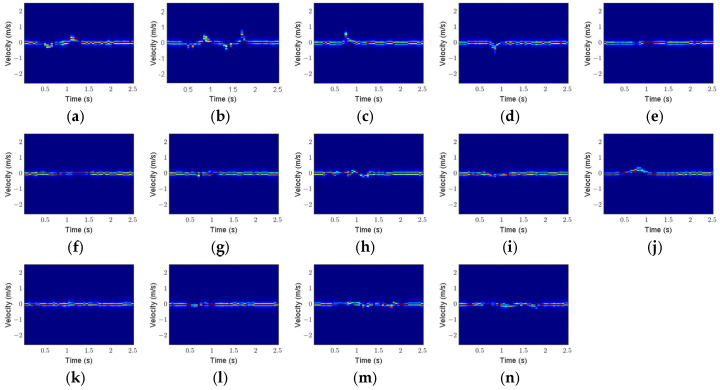
VTMs of 14 gestures after preprocessing: (**a**) click; (**b**) double click; (**c**) beckoning; (**d**) wave; (**e**) sliding left; (**f**) sliding right; (**g**) drawing tick; (**h**) drawing fork; (**i**) palm clench; (**j**) palm open; (**k**) forefinger–thumb open; (**l**) forefinger–thumb close; (**m**) rotating clockwise; (**n**) rotating counterclockwise.

**Figure 22:**
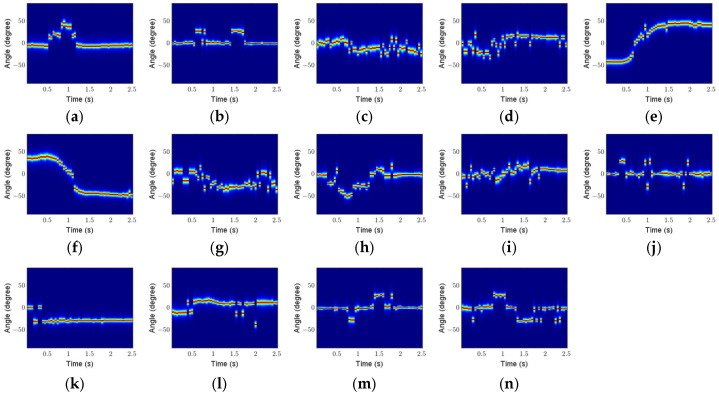
ATMs of 14 gestures after preprocessing: (**a**) click; (**b**) double click; (**c**) beckoning; (**d**) wave; (**e**) sliding left; (**f**) sliding right; (**g**) drawing tick; (**h**) drawing fork; (**i**) palm clench; (**j**) palm open; (**k**) forefinger–thumb open; (**l**) forefinger–thumb close; (**m**) rotating clockwise; (**n**) rotating counterclockwise.

Here, taking Scenario 1 as an example, the feature maps of 14 micro gestures are analyzed. Figure 20, Figure 21 and Figure 22 show the RTMs, VTMs, and ATMs of 14 gestures after preprocessing, respectively.

As shown in Figure 20, the range of the click gesture decreases first and then increases, presenting a V-shaped trajectory. The range of the double-click gesture shows two V-shaped changes. The ranges of the wave and palm clench gestures decrease, while the ranges of the beckoning and palm open gestures increase.

As shown in Figure 21, the velocity of the click gesture first becomes negative, then positive. The velocity of the double-click gesture exhibits two similar changes. The velocities of the beckoning and palm open gestures are positive during the duration of the gesture, while the velocities of the wave, drawing fork, and palm clench gestures are negative during the duration of the gesture.

As shown in Figure 22, the angle of the click gesture is positive during the duration, and the double click gesture shows two similar angle changes. The angle of the sliding left gesture varies from negative to positive, while the angle of the sliding right gesture changes from positive to negative. The angle of the forefinger–thumb open gesture changes from zero to negative, while the angle of the forefinger–thumb close gesture changes from zero to positive. The angle of the rotating clockwise gesture first becomes negative and then positive, while the angle of the counterclockwise rotation gesture changes in the opposite direction.

#### 5.2.3. Feature Fusion Results

**Figure 23:**
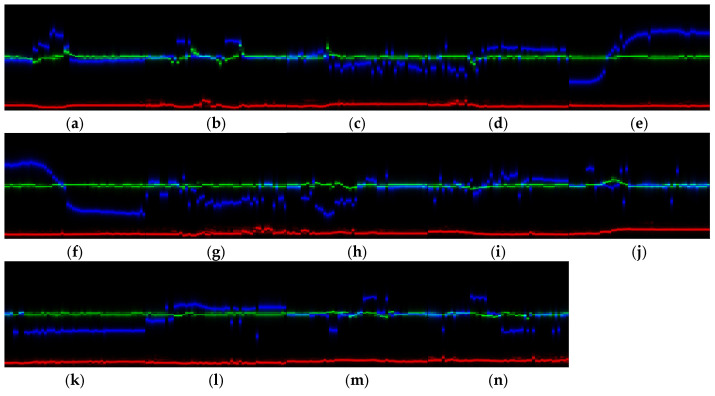
RVATMs of 14 gestures after preprocessing: (**a**) click; (**b**) double click; (**c**) beckoning; (**d**) wave; (**e**) sliding left; (**f**) sliding right; (**g**) drawing tick; (**h**) drawing fork; (**i**) palm clench; (**j**) palm open; (**k**) forefinger–thumb open; (**l**) forefinger–thumb close; (**m**) rotating clockwise; (**n**) rotating counterclockwise.

By fusing the RTM, VTM, and ATM in the color space, an RVATM with a size of 128 × 50 × 3 is obtained. Figure 23 shows the RVATMs of 14 gestures. The red, green, and blue lines represent the trajectories of range, velocity, and angle, respectively.

Compared with the RTM, VTM, and ATM, RVATM contains three-dimensional features, providing a more comprehensive description of motion characteristics, which is beneficial for distinguishing different micro gestures.

After feature extraction, preprocessing, and feature fusion of the collected samples, a dataset containing 8400 RVATMs is obtained, with 600 RVATMs for each gesture.

### 5.3. Recognition Results and Analysis

#### 5.3.1. Evaluation Metrics

In this study, five metrics are used to measure the performance of the proposed network, including accuracy, parameters, computational complexity, model size, and frames per second (FPS).

Accuracy represents the overall recognition performance for all types of gestures, which is given by

(52) $$Accuracy=\frac{TP+TN}{TP+TN+FP+FN}=\frac{1}{N_{total}}\sum_{i=1}^{K}TP_{i}$$

where *TP* represents the number of correctly classified positive samples, *FP* represents the number of incorrectly classified negative samples, *FN* represents the number of incorrectly classified positive samples, *TN* represents the number of correctly classified negative samples, *K* represents the number of types, and *N_total_* represents the number of samples.

The parameters represent the sum of all trainable parameters in the network, reflecting the complexity of the network structure. Computational complexity is assessed through floating point operations per second (FLOPs), which determines the power consumption. Model size indicates the storage requirement of the network. FPS indicates the number of frames processed by the network per second, reflecting the inference speed of the network.

#### 5.3.2. Network Training

The constructed dataset is divided into a training set, validation set, and test set, at a ratio of 6:2:2. The input RVATM is resized to 128 × 128 × 3, the batch size is 16, and the maximum training epoch is 1000. The Adam optimizer is employed for training, and the initial learning rate is 0.001. In addition, the early stop method is used to detect the accuracy of the validation set and terminate the training process when generalization performance begins to decline.

**Figure 24:**
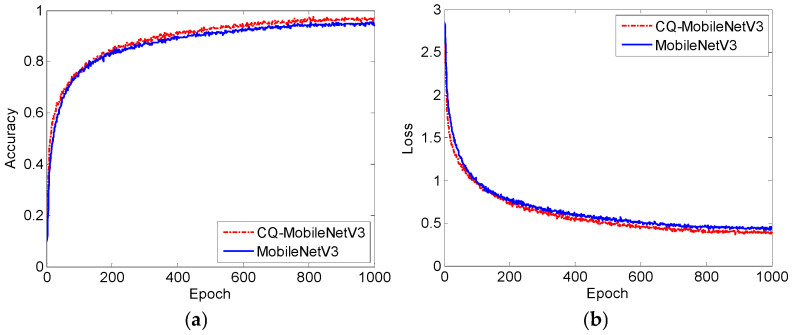
Training curves of MobileNetV3 and CQ-MobileNetV3: (**a**) accuracy curves; (**b**) loss curves.

Figure 24 shows the training curves of MobileNetV3 and CQ-MobileNetV3. As shown in Figure 24, compared with MobileNetV3, CQ-MobileNetV3 achieves faster convergence speed in the first 100 epochs and has slightly higher stable accuracy and slightly lower stable loss.

#### 5.3.3. Ablation Experiments

Ablation experiments are conducted to assess the performance of the three improvements. Table 3 lists the experimental results.

MobileNetV3 is used as the baseline network in the experiments. Network A optimizes the network structure, resulting in a 79.4% decrease in parameter count and a 43.3% decrease in computational complexity. However, the accuracy also decreases by 2.71%. Network B optimizes the network structure and introduces the improved CBAM into the bottleneck blocks, reducing the parameter count by 79.1% and computational complexity by 41.1%, while maintaining almost the same accuracy. After optimizing the network structure, Network C introduces the improved SA module into the bottleneck blocks, reducing the parameter count by 77.9%, the computational complexity by 38.1%, and the accuracy also decreases by 0.62%. The proposed network contains three improvements, which reduce the parameter count by 77.8%, the computational complexity by 37.6%, and increase the accuracy by 0.41%. The results indicate that the three proposed improvements can greatly decrease the parameter count and computational complexity, while enhancing the accuracy.

#### 5.3.4. Comparison with Other Networks

To further evaluate the performance of CQ-MobileNetV3, several state-of-the-art networks were selected for comparative experiments, including DenseNet + CBAM [25], ResNet50 [26], Xception [27], ResNet18 + CBAM [37], GhostNetV3 [38], MobileNetV4 [39], and Swin-Transformer-Small [40].

**Figure 25:**
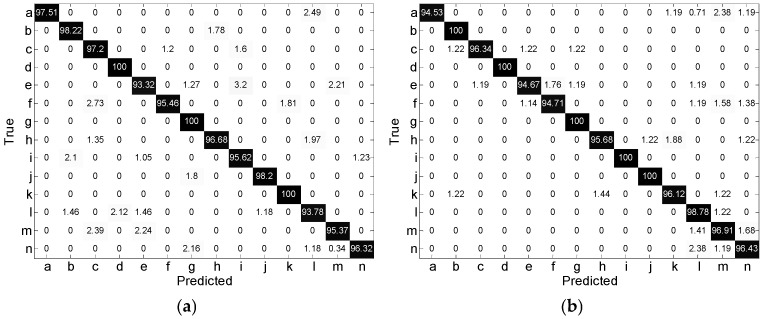

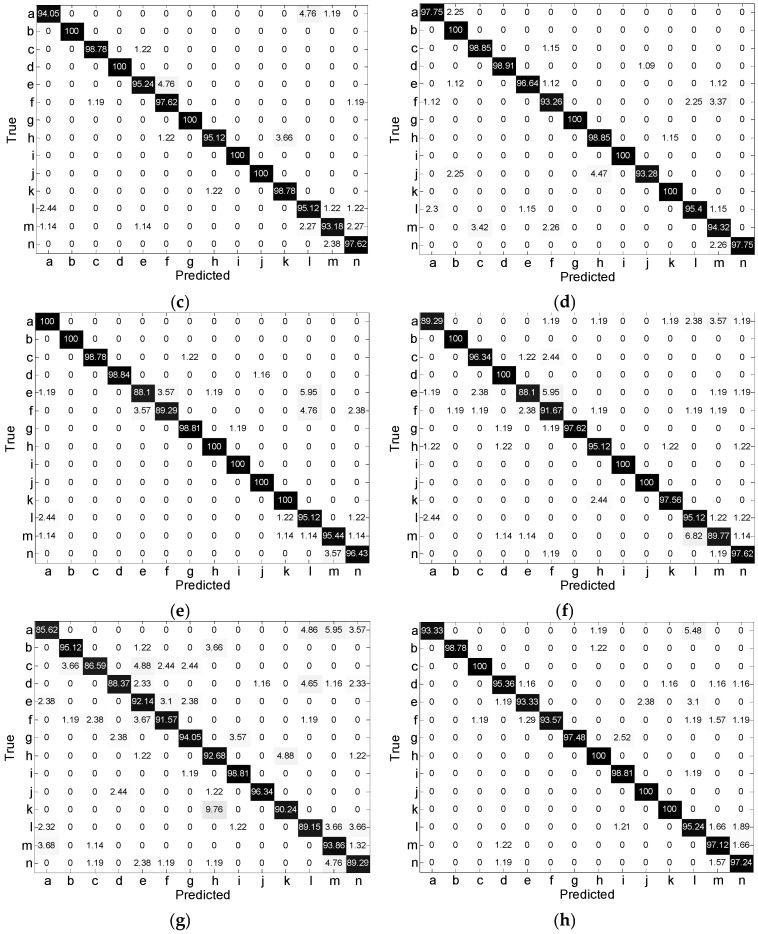
Confusion matrices of eight networks: (**a**) DenseNet + CBAM; (**b**) ResNet50; (**c**) Xception; (**d**) ResNet18 + CBAM; (**e**) GhostNetV3; (**f**) MobileNetV4; (**g**) Swin-Transformer-Small; (**h**) CQ-MobileNetV3.

Figure 25 shows the confusion matrices of the eight networks. The recognition accuracy of a single gesture for all networks exceeds 85%. Except for Swin Transformer Small, the other seven networks achieve 100% accuracy for at least three gestures. These results indicate that the RVATM can effectively depict the motion characteristics of micro gestures and is suitable for different networks.

Table 4 presents a comprehensive performance comparison of the eight networks. As shown in Table 4, ResNet50 and Xception have high accuracy but a high parameter count and computational cost. Swin-Transformer-Small has the highest parameter count but relatively low accuracy. Compared with ResNet18 + CBAM, GhostNetV3 has similar accuracy, with a lower parameter count and computational complexity. MobileNetV4 has much lower parameter and computational complexity than GhostNetV3, but its accuracy is also 1.61% lower than GhostNetV3.

The proposed CQ-MobileNetV3 achieves an accuracy of 97.16%, which is less than 0.5% lower than the highest accuracy. The proposed network has the lowest parameter count and computational complexity, which are only 8.24% and 21.8% of those of the second lowest network (MobileNetV4), respectively. In addition, CQ-MobileNetV3 achieves the highest inference speed of 309 FPS. The results show that CQ-MobileNetV3 maintains high recognition accuracy while implementing lightweight design, obtaining a balance between lightweight design and recognition accuracy through efficient feature extraction networks and attention mechanisms.

## 6. Discussion

Feature extraction and network design are two key stages of radar-based gesture recognition.

In the feature extraction stage, first the three traditional feature maps RTM, VTM, and ATM are extracted by stacking the data of multiple frames. Then, the original RTM, VTM, and ATM are preprocessed to reduce interference and highlight gesture features. Finally, the preprocessed RTM, VTM, and ATM are fused in the color space to construct the RVATM. Compared with the multiple single-dimensional feature maps used in previous studies, the RVATM contains refined multi-dimensional features, which can fully describe the motion characteristics of micro gestures with less information redundancy and avoid further feature fusion processing in the network, thereby reducing the computational complexity of the network.

In the network design stage, achieving a lightweight structure is the main design goal, and the classic lightweight network MobileNetV3-Small is selected as the baseline network. First, based on the size of the RVATM feature map, the network structure is optimized by reducing the number of bottleneck blocks and decreasing the expansion size, which can greatly reduce the number of parameters. Subsequently, two lightweight attention modules, the improved CBAM and the improved SA module, are proposed and integrated into the bottleneck block, constructing the Bottleneck_CBAM and Bottleneck_SA module. Finally, the Bottleneck_CBAM and Bottleneck_SA module are used to replace bottleneck blocks in the structurally optimized network to obtain the CQ-MobileNetV3 network. The two lightweight attention modules can effectively improve the recognition accuracy, while increasing little computation complexity and parameter count.

In the experiment, using the RVATM as the input feature map, different networks achieve recognition accuracy above 90% for 14 types of micro gestures, demonstrating the good adaptability of the RVATM. Compared with several other mainstream networks, CQ-MobileNetV3 significantly reduces the parameter count and computational complexity, and achieves the highest inference speed while maintaining high recognition accuracy. These results indicate that CQ-MobileNetV3 effectively balances network lightweight design and recognition accuracy, making it suitable for deployment on mobile devices with limited computational and storage resources.

This study has some limitations. First, only scenarios where the hand is at a relatively close distance above the radar are studied. The performance of the proposed network may degrade in scenarios where the hand is at longer distances and different directions relative to the radar. Second, feature extraction in complex backgrounds with more interference also needs to be considered.

## 7. Conclusions

In this study, a micro gesture recognition method using multi-dimensional feature fusion and a lightweight network is presented. In feature extraction, the RTM, VTM, and ATM are first extracted from raw data and refined through preprocessing. Then, the three feature maps are fused in color space to obtain the RVATM, which can well express the motion information of gestures. For recognition, a lightweight CQ-MobileNetV3 is presented. First, the redundant parameters and computation are reduced by optimizing the network structure. Then, the recognition accuracy is improved by integrating the improved CBAM and the improved SA module. The experimental results based on the 77 GHz FMCW radar show that the CQ-MobileNetV3 network obtains a high accuracy of 97.16% for 14 micro gestures, with a parameter count of 0.207 M, a computational complexity of 0.027 GFLOPs, and a model size of 0.895 MB. The results validate that the proposed network is superior to seven other networks in terms of comprehensive performance.

In future work, interference suppression techniques in feature extraction against complex backgrounds will be studied. In addition, the improvement of the network will be investigated to enhance recognition performance in different scenarios.

## Figures and Tables

**Table 1:** Optimized network structure.

Module	Expansion Size (Original/Optimized)	Output Size
Bottleneck_SE	16/16	64 × 64 × 16
Bottleneck	72/16	32 × 32 × 16
Bottleneck_SE	88/72	16 × 16 × 24
Bottleneck_SE	144/96	8 × 8 × 48
Bottleneck_SE	288/144	8 × 8 × 48
Bottleneck_SE	576/96	4 × 4 × 96
Bottleneck_SE	576/192	4 × 4 × 96
Conv2d, 1 × 1		4 × 4 × 576
Pool		1 × 1 × 576
Conv2d, 1 × 1		1 × 1 × 14

**Table 2:** Parameter configuration of the radar system.

Parameter	Value
Number of transmitting antennas	2
Number of receiving antennas	4
Starting frequency	77 GHz
Modulation slope	100 MHz/us
Chirp period	380 us
Chirp duration	40 us
Number of chirps per frame	128
Frame period	50 ms
Sampling rate	2 MHz
Samples per chirp	64
Number of frames	50

**Table 3:** Results of ablation experiments.

Network	Structure Optimization	Improved CBAM	Improved SA	Accuracy (%)	Params (K)	FLOPs (M)	Size (KB)	FPS
MobileNetV3				96.75	932.96	42.99	3786	270
A	√			94.04	192.49	24.36	814	331
B	√	√		96.32	194.63	25.31	823	320
C	√		√	96.13	206.64	26.59	864	315
Proposed	√	√	√	97.16	207.24	26.81	895	309

**Table 4:** Comprehensive performance comparison of different networks.

Network	Accuracy (%)	Params (M)	FLOPs (G)	Size (MB)	FPS
DenseNet + CBAM	96.98	7.012	1.924	28.025	182
ResNet50	97.44	23.537	2.698	89.785	199
Xception	97.54	20.836	2.982	78.482	281
ResNet18 + CBAM	97.50	11.346	1.193	44.496	124
GhostNetV3	97.20	8.129	0.300	31.010	158
MobileNetV4	95.59	2.511	0.124	9.579	381
Swin-Transformer-Small	91.70	49.800	17.089	191.399	35
CQ-MobileNetV3	97.16	0.207	0.027	0.895	309

## Data Availability

The data presented in this study are available upon request from the corresponding author.
